# Intrinsically disordered proteins and membranes: a marriage of convenience for cell signalling?

**DOI:** 10.1042/BST20200467

**Published:** 2020-11-06

**Authors:** Jasmine Cornish, Samuel G. Chamberlain, Darerca Owen, Helen R. Mott

**Affiliations:** Department of Biochemistry, University of Cambridge, 80, Tennis Court Road, Cambridge CB2 1GA, U.K.

**Keywords:** intracellular signaling, intrinsically disordered proteins, membrane dynamics

## Abstract

The structure-function paradigm has guided investigations into the molecules involved in cellular signalling for decades. The peripheries of this paradigm, however, start to unravel when considering the co-operation between proteins and the membrane in signalling processes. Intrinsically disordered regions hold distinct advantages over folded domains in terms of their binding promiscuity, sensitivity to their particular environment and their ease of modulation through post-translational modifications. Low sequence complexity and bias towards charged residues are also favourable for the multivalent electrostatic interactions that occur at the surfaces of lipid bilayers. This review looks at the principles behind the successful marriage between protein disorder and membranes in addition to the role of this partnership in modifying and regulating signalling in cellular processes. The HVR (hypervariable region) of small GTPases is highlighted as a well-studied example of the nuanced role a short intrinsically disordered region can play in the fine-tuning of signalling pathways.

## Introduction

The discovery and characterization of intrinsically disordered proteins and regions (IDPs and IDRs) shattered the long-held structure-function paradigm. Disordered proteins exist in a population of different states, with their structures being best described by an ensemble of conformations. The structured, ordered proteins fold by finding a deep minimum within the energy landscape that they sample while folding. In contrast, the energy landscape of disordered proteins is characterized by multiple, shallow minima, which correspond to a number of different states that the protein can sample without energetic penalty. They fluctuate rapidly through these different states and this structural plasticity is integral to their function [1,2]. Structural disorder is characterized by low sequence complexity, high net charge and a lack of hydrophobic residues. Based on this set of characteristics, it is calculated that >20% of human proteins contain regions of disorder longer than 30 residues [3–5].

The same sequence characteristics that are found in disordered regions are also utilized for interactions with lipid bilayers. The prevalence of charged side chains in IDRs allows electrostatic interactions with charged lipid head groups [6–8] and therefore membrane composition affects the binding of IDRs (Box 1). In addition, proteins that associate transiently with membranes must also be stable when not membrane bound and so they are required to be conformationally versatile. Although there are few hydrophobic amino acids in disordered regions, those that are present have solvent exposed side chains and are therefore poised to be buried. Consequently, lipid-binding proteins account for 15% of all disordered proteins [9,10].

Membrane-bound organelles bring many benefits to a multicellular organism. Compartmentalization allows discrete control over specific processes and preserves environmental properties, such as pH and local concentration of substrates. The membranes that surround organelles can be modified and used as anchors and hubs for signalling cascades. Disordered proteins and regions are found throughout cell signalling, but in relation to membranes, they are particularly prevalent in the cytoplasmic tails of transmembrane receptors, as mediators of protein–protein networks and in vesicle trafficking and membrane modulation assemblies [11,12]. This review will discuss the principles that drive IDRs to bind and/or modulate membranes and will summarize the relationship between membrane-associating IDPs/IDRs and signalling across a wide range of cellular processes. It will then focus on the rapidly evolving field of the HVRs (hypervariable regions) of small GTPases, which have been the subject of detailed investigations into the effects of a disordered region on the regulation and modulation of signalling output.

## Principles of disorder in membrane binding

### Curvature sensing

**Figure 1.**
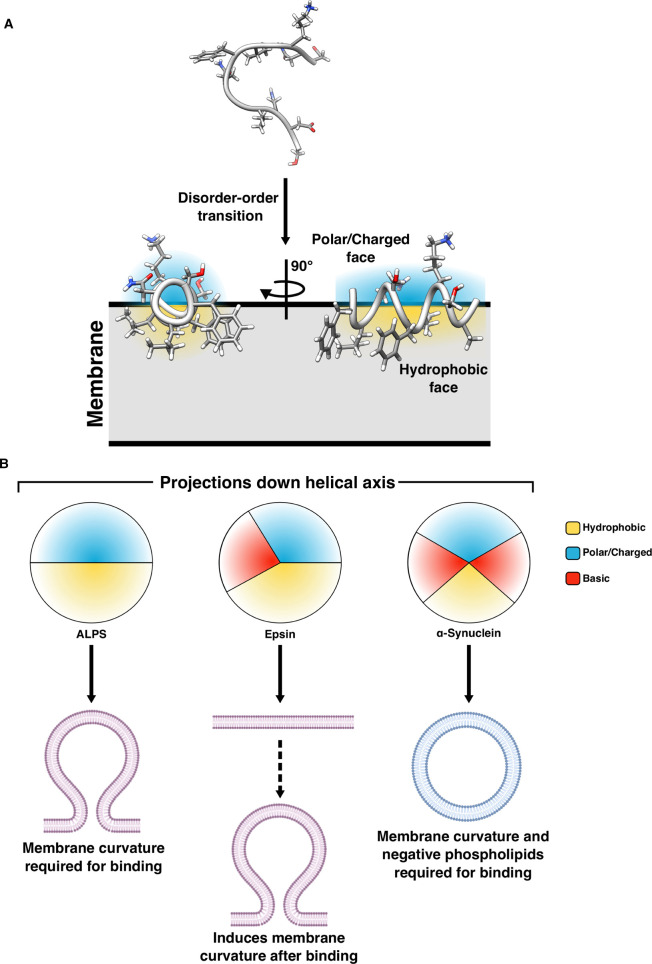
Folding of disordered regions into amphipathic helices. (**A**) Disordered regions can fold upon membrane binding, forming amphipathic helices, with the hydrophobic face adsorbed into the membrane and polar/charged residues exposed to the lipid heads and the aqueous environment. The helices lie along the plane of the membrane. (**B**) Projection down the helical axis of a representative ALPS motif, Epsin and α-Synuclein. As membrane binding is driven solely by hydrophobic interaction, the ALPS motif requires membrane curvature for binding. Epsin has a biased charge distribution to enable PIP2 specific binding. Once bound Epsin can induce membrane curvature. Due to the limited hydrophobic face of α-Synuclein, electrostatic interactions are also required to stabilize the binding to curved membranes.

Some IDRs become ordered upon binding to other molecules and the appearance of structure can arise from the capture of one member of the ensemble (conformational selection), which shifts the equilibrium of ensemble structures towards the ordered state, or folding may be induced by binding (induced fit) [2,13,14]. Disorder-to-order transition due to membrane binding usually induces folding into amphipathic helices where the hydrophobic and polar residues are segregated across the two faces of the helix (Figure 1A). Membrane-adsorbing helices lie parallel along the membrane, with their hydrophobic side chains buried and their polar/charged face exposed to the cytosol and to the phospholipid headgroups [15,16]. Both hydrophobic and electrostatic interactions contribute to binding: hydrophobic residues insert into the lipid bilayer while polar residues can push apart adjacent phospholipid headgroups. When positively charged side chains on the polar face interact with negatively charged phospholipids, this can cause membrane bending (Figure 1B). However, if charged polar residues are not present in the helix, then high membrane curvature is required for membrane adsorption. In curved membranes, the phospholipid headgroups are already spread apart so that the interaction can be driven solely by the hydrophobic effect [17–20]. Disordered proteins peripheral to the membrane can also sense membrane curvature through an entropic mechanism. The membrane constrains the disordered region, reducing its conformational entropy. As a membrane becomes more curved, this constraint is reduced. Therefore, the disordered region has a preference for curved membranes, where its conformational entropy is higher. This is exemplified by Epsin, which will be discussed later in its biological context. If there is high net negative charge within the disordered domain, there is also electrostatic repulsion of anionic lipids, which also contributes to curvature sensing [21–23].

N-BAR containing proteins provide an example of membrane-curvature sensing by an IDR. N-BAR regions are homodimers, consisting of a disordered N-terminus plus a helical, dimeric BAR domain and are present in amphiphysin, endophilin and nadrin (a neuron-specific GAP), amongst others. The disordered N-terminus of N-BAR regions folds into an amphipathic helix when negatively charged phospholipids are present. Together the BAR domain and the N-terminal amphipathic helix co-ordinate to sense curvature and cause membrane bending and tubulation. The N-terminal region binds to and folds on curved membranes, which ensures close association of the BAR domain to the membrane. The BAR domain dimer then drives curvature via its curved helices [19,24,25].

### Localization

Localizing proteins to membranes increases the number of complexes that can be formed by increasing the effective concentration of their components in a small volume. Interactions between membrane-bound proteins are effectively confined to a 2D space, rather than three dimensions in the cytosol. Reducing the dimensionality through membrane binding limits the necessary search space of the protein, and thereby increases their collision probability [26–28]. As well as increasing the effective concentration, membranes also act as a steric barrier, that restrict interactions that could be possible in solution. Disordered regions of different lengths can therefore act to tether proteins at the appropriate distance from the membrane to interact with specific partners [29].

Despite the obvious advantages, this raises the problem of getting these proteins to a membrane. The large space sampled by disordered regions can overcome this by acting as a wide net to encounter binding partners, in a mechanism that has been termed fly-casting [30,31]. The combination of membrane association and a large capture net means that IDRs can recruit proteins to a variety of membranes including the plasma membrane and organelles [26,32]. It should be noted however that although often cited, the benefits of fly-casting have been disputed. Initially, it was thought that large capture nets increase the association rates of IDRs over those of folded proteins. However, this has been hard to prove experimentally i.e. by changing the amount of disorder in these proteins, as this affects more than just their association rates. Furthermore, when IDPs that have the propensity to fold are stabilized, their association rates are usually *increased*, rather than decreased, presumably due to a lower entropic penalty of binding. Huang and Liu instead suggested that extended proteins have more encounters with binding partners, leading to more productive interactions [31,33]. Despite discrepancies in our understanding of the mechanism, the term fly-casting is used liberally to describe how IDRs, with their increased interaction range, can capture proteins from the surrounding environment, whilst being restricted in their mobility.

### Post-translational modifications

IDPs are well suited to host post-translational modifications (PTM) due to their solvent-exposed, reactive side chains and extended surfaces that are readily accessible to kinases and other modifying enzymes [34–36]. Phosphorylation is a well-studied PTM that can modulate both membrane binding and intracellular signalling cascades. For example, the introduction of additional negative charges can disrupt electrostatic interactions with the membrane [37], can affect backbone dynamics and push the population towards one particular state or can cause folding of disordered regions [2,38–40]. Other types of PTM commonly found in IDPs such as glycosylation, methylation and lipid modifications can also modulate the function of IDPs but are often permanent modifications [27,33]. Of these, lipid modifications are involved in regulatable membrane-association. Post-translational addition of a lipid moiety may be used to anchor proteins to membranes, often in addition to basic residues [41–43].

MARCKS protein is a good example of how PTMs can modulate membrane-association. MARCKS associates with the plasma membrane via an N-terminal myristoyl moiety in combination with a polybasic region in the middle of the protein. Phosphorylation of the polybasic region is thought to act as a simple electrostatic switch, whereby reducing the net charge reduces membrane interactions [8,37,44]. Another protein with a myristoyl and polybasic membrane-binding domain is c-Src [8,44]. NMR investigations into this region provide more insight into the general mechanism of polybasic membrane binding. The complex remains ‘fuzzy’ when membrane bound — it adopts a compact but highly dynamic conformation. More detailed investigations need to be carried out to establish if this is a common trait between polybasic membrane binders. As with MARCKS, phosphorylation close to the membrane binding sites of c-Src electrostatically destabilizes the membrane interaction. However, phosphorylation of c-Src has also been shown to affect the compaction of the disordered region, which alters the conformational restriction of the fuzzy complex [45–47].

### Phase-separation and clustering

Phase separation is the condensation of protein, protein–DNA or protein–RNA complexes into liquid droplets. In a process often likened to the behaviour of oil in water, the droplets form discrete compartments or membrane-less organelles. Multivalent molecules, such as IDPs, which have multiple interaction sites, naturally assemble, which then can drive phase separation as a result of decreased solubility. Although phase separation is not restricted to IDPs, it is a common feature, as their low-complexity amino acid composition lends itself to phase transition and their flexibility promotes a liquid, disordered state. The reasons why low-complexity regions cause phase separation are still to be elucidated but hypotheses tend towards imperfect sequence repeats allowing multiple, weak intra- and intermolecular interactions with the type of interaction varying between IDRs. Hydrophobic interactions are a common feature, and aromatic stacking has been shown to drive phase separation and stabilize phase-separated structures. Additionally, IDRs with high net charge have been shown to phase separate based on complementary electrostatic interactions with other proteins, forming complex, multi-component coacervates [48–51]. Classical examples of phase-transitioned condensates include the nucleolus, stress granules, P-bodies and Cajal bodies [52]. As an extremely active area of research, terms relating to this phenomenon include both phase-transition and clustering, with overlapping usage. Clustering is often used to describe the recruitment of multiple copies of a protein to a microenvironment at the membrane surface and includes recruitment driven by protein–membrane interactions. It is also sometimes used to describe cases where phase-transition has not yet been shown experimentally. Phase separation is a physical process driven by low affinity, high avidity protein–protein/protein–nucleic acid interactions. Phase separation at the membrane occurs in an approximately 2D space and is driven by cytoplasmic protein regions.

Transmembrane signalling is currently the most active area of research with regards to phase-transition at the membrane, with less examples of peripheral membrane-bound phase-separating IDPs. However, with the abundance of IDRs found at membranes, it is unlikely that this will remain the case [53–55]. The utility of phase separation in cellular signalling has been well-reviewed recently [52]. Phase separation of membrane bound IDPs leads to amplification of signals by increasing the local concentration of components. It can also regulate pathways through selective inclusion/exclusion of signalling components and, in some instances, this may even extend to switching on or off particular branches of a pathway [52].

Layered onto the concept of phase separation of proteins on the membrane surface is that the membrane lipids themselves are able to cluster into so-called ‘lipid rafts’. These are small regions of the membrane that are enriched in saturated lipids and cholesterol, driven by preferential interactions between lipids. They also undergo liquid–liquid phase separation that could be driven by, or that modulate, phase separation or clustering of membrane-binding proteins. A detailed discussion of lipid rafts is outside the scope of this review, but we refer the reader to two recent reviews of the field [56,57].

## Cellular processes mediated by disordered regions binding membranes

### Signal transduction across membranes

Transmembrane receptors often have intrinsically disordered regions within their cytoplasmic component [58,59] and these cytoplasmic tails are important in transducing the external signal across the membrane. In some cases, these tails make contact with the inner leaflet of the plasma membrane. Evidence of membrane-associating tails has been seen for a range of transmembrane proteins including EGFR [60–62], BAI1 GPCR [63], T cell CD3 receptors [64], CD4 MHC Class II co-receptors [65], human prolactin receptor and growth hormone receptor [66]. Although the mechanistic role of all of these membrane-associating tails is not yet fully understood, there is evidence to suggest that disorder is instrumental for their signal transduction.

**Figure 2.**
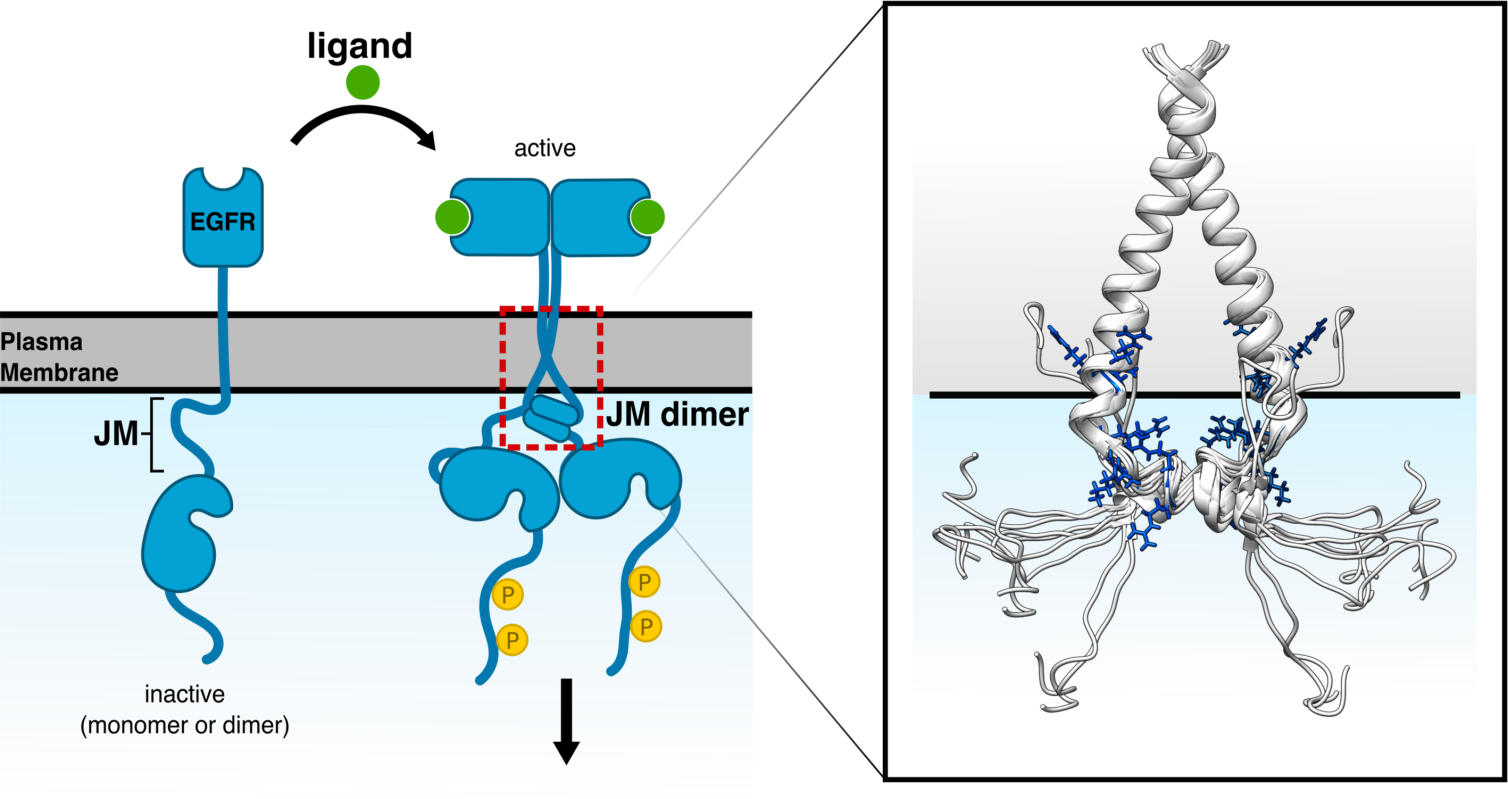
Structural rearrangement and membrane binding of the juxtamembrane (JM) region of the EGFR. The disordered JM of the EGFR contacts the membrane. On growth factor binding, EGFR dimerizes and the juxtamembrane domains fold into helices that dimerize asymmetrically, which leads to asymmetric dimerization of the kinase domains and autophosphorylation (phosphate as yellow circles), which activates downstream signalling. The red box highlights the area shown as the ensemble structure of the ten lowest energy models (grey, PDB 2M20) of the transmembrane and JM regions (right). Residues Arg645-7, Arg651, Lys652 Arg653, Arg656 and Arg657 (blue, sticks) in the JM contact PIP_2_ in the membrane (solid black line illustrates the level of the lipid heads).

Signals can be passed across the membrane through conformational changes in the receptor upon ligand binding. Consequently, ligand binding can cause membrane-associated disordered regions to be released and made available for protein–protein interactions. This is exemplified by EGF receptor tyrosine kinases, where the juxtamembrane (JM) region between the transmembrane α-helix and the kinase domain is flexible with unstable helical regions. In the absence of ligand, basic residues within the JM region contact the membrane [61,62,67]. Membrane imbedded JM regions are compatible with both EGFR monomer and inactive (ligand-free) dimer conformations. In ligand bound, active EGFR dimers, the JM is released from the membrane and formation of an antiparallel dimer with the JM region on the adjacent receptor. Release of the JM from the membrane arises from a rearrangement of the transmembrane helices in the ligand-bound dimer [62,68,69]. The antiparallel JM dimer stabilizes asymmetrical dimerization of the intracellular region, which in the EGFR leads to activation of the kinase domain (Figure 2) [62,70]. Signal transduction is therefore regulated, as membrane interaction prevents dimerization of the JM in the absence of the extracellular signal. It is thought that the overall flexibility of the JM region facilitates the switching between the two states and allows the asymmetrical dimerization of the kinase domains. The disordered JM and the interaction of its basic residues with phosphatidylinositol 4,5-bisphosphate (PIP_2_) also leads to EGF receptor clustering in the plasma membrane. This PIP_2_-dependent clustering facilitates downstream signalling, as dimerization is favoured when the receptors are in close proximity [71]. Due to its multivalent nature, PIP_2_ is able to interact with multiple residues in the JM region, as highlighted in Figure 2. Interaction of the JM with PIP_2_ lowers the free energy of JM-dimerization compared with interactions with less negative lipids such as phosphatidylserine (PS), by increasing the affinity of the dimer for the membrane. Finally, phosphorylation of Thr654 decreases the affinity for the membrane and therefore destabilizes dimer formation [67,70].

Two components of the T-cell receptor complex, namely the ζ chain and CD3ε (co-receptor), have disordered cytoplasmic tails that contain ITAMs (immunoreceptor tyrosine-based activation motifs). Tyrosine residues in ITAM motifs are phosphorylated upon receptor activation and are necessary for downstream signalling events. There is evidence that the side chains of tyrosines in the disordered tails are buried in the membrane. Some helicity is induced when CD3ε and ζ are incubated with small bicelles (disc-like membrane mimetics) but they remain unstructured when bound to larger liposomes (model membranes comprising spherical vesicles) [64,72,73]. Upon extracellular ligand binding, the ensuing conformational changes release the tyrosines from the membrane, allowing them to be phosphorylated by Lck. Phosphorylated tyrosines cannot reassociate with the membrane and so remain accessible for SH2 domain binding and propagation of the signal downstream [73,74]. The disordered tails and membrane therefore co-operate to form the inhibited state, which is quickly and efficiently reversed through the release of the tyrosines from the membrane, also exemplifying how phosphorylation modulates signal transduction by disordered regions [72,73].

T-cell receptor LAT adaptors have been shown to phase separate. LAT has a transmembrane region and an intracellular IDR that promotes phase separation of itself and T-cell receptor complexes. Kinases are also sequestered into the phase-separated space and phosphatases are excluded, prolonging the action of the external signal. It is becoming apparent that phase separation is a common mechanism of increasing the local concentration of receptors and may be the driver for what had previously been described as receptor clustering [53,54].

Cytokine receptors have similar motifs to ITAMs within their disordered cytoplasmic regions, which also bind the plasma membrane as shown by their *in vitro* preference for lipids found in the inner leaflet. However, in contrast with ITAM motifs, the intracellular regions of the human prolactin receptor and growth hormone receptors do not fold upon lipid binding and their binding to lipids is not modulated by phosphorylation [66,75]. The role of lipid binding by the intracellular ITAM-like motifs is yet to be elucidated but two models have been proposed for the transmission of the signal to the intracellular regions when ligand interacts with the extracellular domain. The first proposes that ligand binding, at least for the growth hormone receptor dimer, causes the intracellular regions of the pre-dimerized receptor to move away from each other along the membrane plane. Each receptor monomer is bound to one copy of the effector kinase JAK2, which is inhibited in *trans* by binding to a pseudosubstrate motif on the JAK2 bound to the other receptor monomer. The conformational change induced on ligand binding rearranges the JAK2 dimer so that the pseudosubstrate no longer blocks the active site and an active dimer is formed. The second model proposes that the conformational change in the transmembrane domain causes rearrangements in the lipids of the inner plasma membrane, which drive the changes in the intracellular regions and bound JAK2 proteins. Due to the flexible nature of the intracellular region, most experimental data has focussed on the transmembrane and extracellular domains and therefore supports either model [76,77].

### Scaffolding of signalling complexes

The extended nature of disordered proteins means that they can function to co-ordinate multiple binding partners and act as signalling hubs and scaffolds. Signalling hubs are able to interact with many different proteins, forming transient contacts between only a few proteins at a time and therefore potentially acting as temporal organizers [78]. Scaffolds are, instead, able to make multiple contacts simultaneously, facilitating contacts between the proteins that they bind [79–81]. In this way IDPs act as convergence points with different properties, activating multiple signalling pathways according to need [82–84].

The addition of a membrane-binding region enables scaffolding proteins to localize their multiple interactions to the correct space. AKAPs (A-kinase anchoring proteins) are disordered scaffolding proteins with three membrane binding polybasic regions and are often myristoylated (Box 1) at their N-termini [85]. Membrane binding and subsequent localization of AKAP12 to the cell periphery allows scaffolding of β-adrenergic receptors, protein kinases A and C (PKA and PKC) and protein phosphatase 2B with GRK2 (G protein-coupled receptor kinase). The increased proximity of PKA and GRK2 to the receptor leads to phosphorylation and therefore desensitization of the receptor. The phosphorylated receptor has enhanced binding to β-arrestin, which recruits clathrin and leads to sequestration of the entire complex [41,86].

Basic residues that bind anionic lipids are also seen in the disordered tail of the NMDA receptor. The disordered tail of this receptor shares properties with AKAP proteins: it acts as a scaffolding region, is phosphorylated by PKC and has been shown to bind Calmodulin [7,87]. Unlike AKAP however, NMDA is a transmembrane protein, so membrane attachment is not driven by its disordered tail. Rather, it is possible that lipid specificity causes lateral movement and clustering of multiple receptors [88,89].

### Vesicle trafficking

Amphipathic helices that are able to sense membrane curvature are often utilized in vesicle trafficking. Amphipathic lipid packing sensor (ALPS) motifs, originally identified in ArfGAP1, are the most well studied of these bi-faced helices [17,90]. The ALPS motifs in ArfGAP1 fold in the presence of curved membranes or sparsely packed membranes (Figure 1B), with serine and threonine making up the bulk of the polar residues. ArfGAP1 uses its ALPS motif to sense vesicle budding induced by COPI coat proteins on Golgi membranes, coupling curvature sensing with enzymatic activity. It is thought that the high curvature of budding vesicles recruits ArfGAP1, where it is then co-localized with Arf1. COPI coat proteins, which surround the budding vesicles, are also necessary for ArfGAP1 activity, which then facilitates the hydrolysis of GTP to GDP on Arf1·GTP, switching off Arf1, leading to COPI disassembly only on the fully formed vesicles [20,90–92].

The synaptic vesicle protein, α-synuclein, is also thought to act as a membrane curvature sensor. When bound to membranes, the disordered N-terminus folds into one of two possible conformations, one long amphipathic helix or a broken, antiparallel helical pair [93–95]. The long helix forms on surfaces with a curvature similar to that of a synaptic vesicle, whereas on highly curved membranes, only the broken helix is able to form, as smaller vesicles cannot support the length of the long helix [96]. A balance between the ordered and disordered states allow α-synuclein to interact simultaneously with two membranes and so it may play a role in vesicle fusion [97]. Alpha-synuclein is thought to play a role in the synaptic vesicle cycle, which is likely to utilize this ability to sense membrane curvature to discriminate between curved vesicles and flat membranes [98,99]. Alpha-synuclein requires both curvature and the presence of negatively charged lipids to bind to membranes (Figure 1B). Along the amphipathic helix, the boundary between the hydrophobic and polar faces is rich in lysine residues, while the hydrophobic face contains only small hydrophobic residues such as alanine and valine. The small residues in the hydrophobic face do not provide a strong enough hydrophobic anchor for it to bind to neutral membranes, even if curved. Instead, the membrane interaction is stabilized by electrostatic interactions between the lysine residues at the interface and negative phospholipids [93,96,100,101].

**Figure 3.**
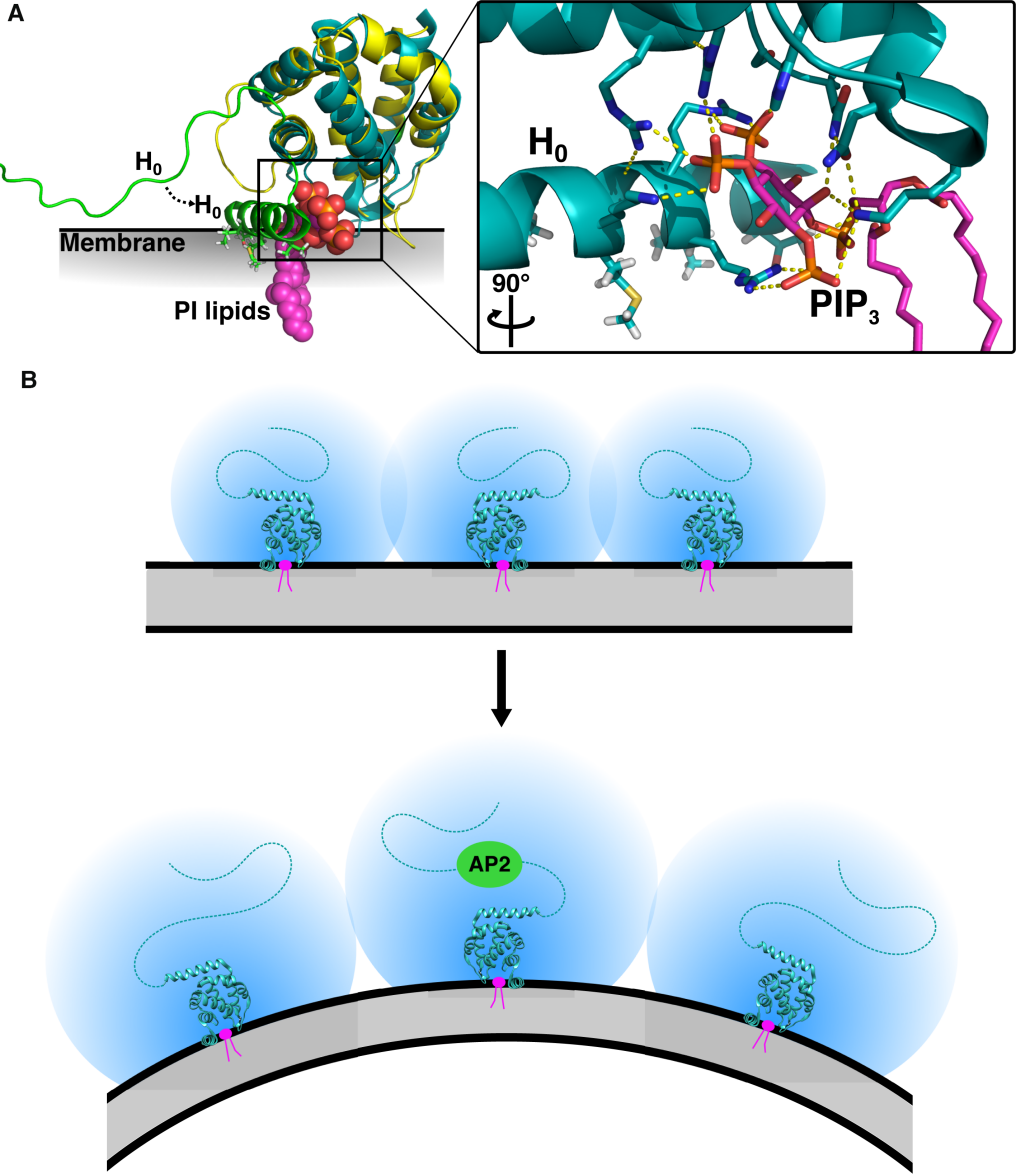
Membrane bending induced by Epsin. (**A**) Structural comparison of ENTH with (teal, PDB 1H0A) and without (yellow, PDB 1INZ) a phosphoinositol (PI) lipid. In the presence of PI (pink), H_0_ of ENTH folds (shown in green for both states) and inserts into the lipid bilayer, forcing a wedge between the lipids. Residues in H_0_ and additional residues in the ENTH domain form a hydrogen bonding network (shown inset with dashed yellow lines) to contact the PI headgroup. (**B**) Membrane bending is thought to be caused by steric pressure between the disordered C-terminal regions of multiple copies of Epsin. Top: The overlapping spheres represent the radius of Epsin and show the steric clashes between the disordered regions when the bilayer is flat. Bottom: AP2 binding has been shown to increase the radius of Epsin, which would induce greater curvature by pushing membrane-anchored Epsin molecules further apart.

Amphipathic helices containing positively charged side chains are usually involved in vesicle budding, membrane remodelling and fission rather than curvature sensing. Epsin recruits accessory proteins for clathrin-mediated endocytosis and consists of a partially-ordered ENTH domain, which binds to membranes, and a disordered C-terminus. The first amphipathic helix (H_0_) of the ENTH domain is also disordered in solution but folds on binding to inositol phospholipids (specifically PIP_2_, with weaker binding seen for other inositol phospholipids that have a phosphate at both positions 4 and 5 on the inositol ring) and is inserted into the bilayer. Inositol binding is required for correct targeting of Epsin to the plasma membrane. H_0_ mutants, which no longer binding to PIP_2_ *in vitro*, are cytosolic *in vivo* [102,103]. In addition to contacts made by H_0_, membrane binding is stabilized by the interaction of side chains in the ENTH domain, which make non-specific electrostatic interactions with lipid phosphate groups (Figure 3A). Insertion of H_0_ into the plasma membrane deforms the bilayer, prior to clathrin-mediated endocytosis. Recent work has shown that Epsin can tubulate membranes using its disordered C-terminus, as long as it is membrane attached, by a mechanism in which multiple copies of the bulky disordered region induce membrane curvature through steric pressure. Multimers of Epsin combine to drive membrane curvature in simulations [103,104]. A recent study has hypothesized that in response to binding the clathrin adaptor AP2, the radius of the Epsin disordered region expands, further inducing curvature (Figure 3B) [105]. Both mechanisms may combine to drive curvature [22], while the side chains of H_0_ also determine lipid specificity.

Disordered regions are also present in golgins, tethering proteins associated with the Golgi apparatus (reviewed recently in [106,107]). The golgin GMAP-210 senses membrane curvature in order to sort transport vesicles at the entrance of the Golgi. It filters vesicles by size and lipid-packing, utilizing an ALPS motif with infrequent hydrophobic residues, which then insert between lipids that are loosely packed [101,108]. Outside of their N-terminal vesicle-binding regions, golgins have a mainly coiled-coil structure but local unwinding of the coiled-coil is predicted to give regions of flexibility that can collapse and shorten the overall golgin protein. In GMAP-210, these flexible regions have also been shown to interact with Rab GTPases on the vesicle. Interaction with the flexible regions would bring the Rab-marked vesicle into closer proximity to the Golgi membrane, and together with structural collapse, facilitate the transition from the long range ALPS-tethered state, to downstream fusion with the Golgi membrane [109,110].

### Mitochondrial fission

**Figure 4.**
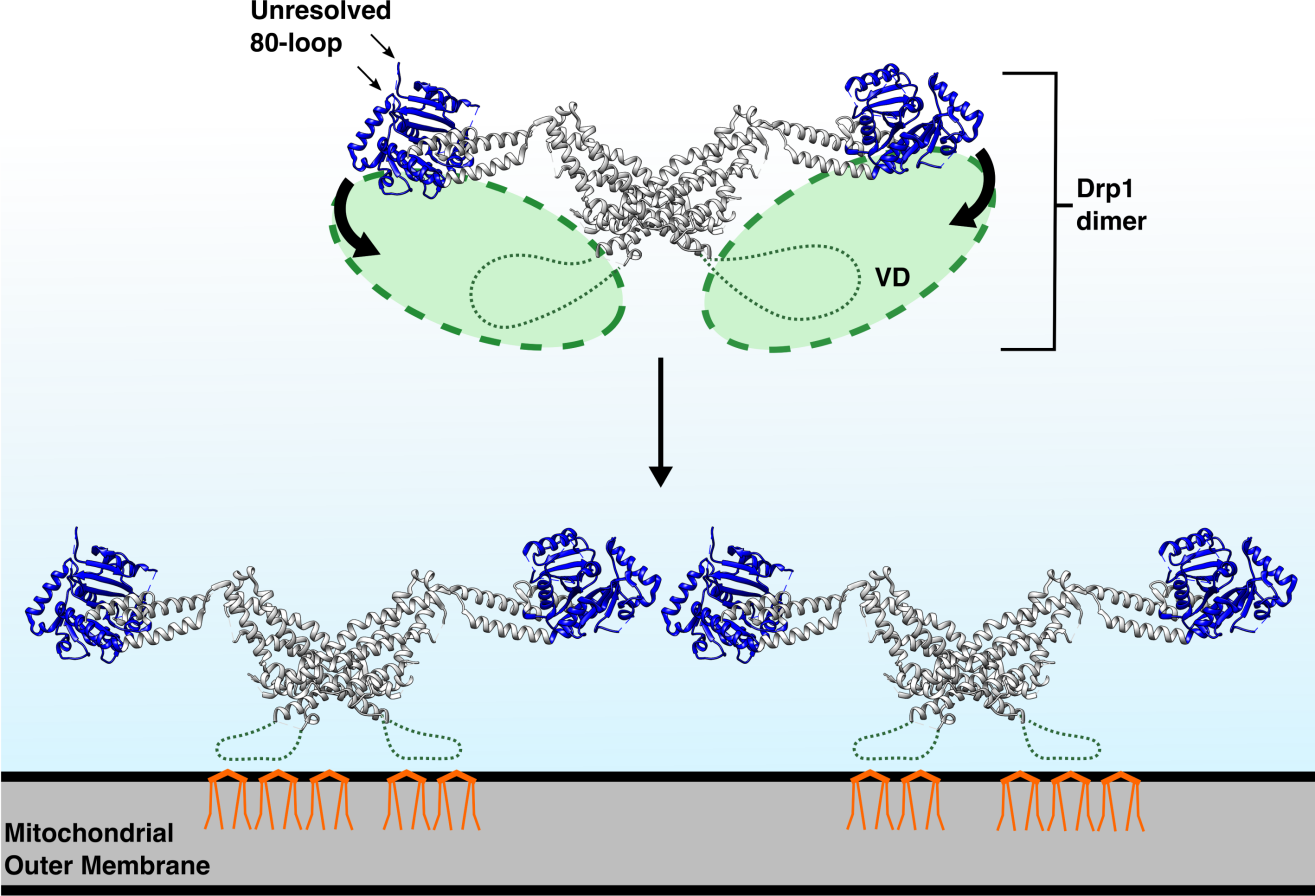
Model of oligomerization of Drp1 dimers on cardiolipin (CL) membranes. In the cytosol, the dynamic variable domain (VD) (green, dashed loop and oval to indicate sampling space) sterically interferes with the dynamin-type G-domain (blue) of Drp1 cytosolic dimer (PBD 4BEJ). Once the VD is restricted by binding to CL in the membrane, the G-domains are free to dimerize, causing the formation of higher-order Drp1 mulitmers. CL lipids (orange) are spatially sorted by the VD. Based on data in [114].

The large GTPase Drp1 (Dynamin related protein 1) is responsible for mitochondrial (and peroxisomal) membrane fission in response to its phosphorylation by CyclinB1/CDK1. It has also been implicated in the scission of endocytic vesicles in the synapse [111,112]. There are two disordered regions in Drp1: the first is the so-called 80-loop, named after its central residue, which is an 18-residue insertion that protrudes from the middle of the G-domain (GTPase-domain); the second is called the variable domain (VD). In the cytosol, the VD is dynamic and inhibits dimerization of the G-domain, which prevents GTPase activity. The VD binds weakly, but preferentially, to cardiolipin (CL) present in mitochondrial membranes (Figure 4). The specific residues and mechanism of the interaction between VD and CL are yet to be elucidated but once membrane bound, the VD is constrained. This allows oligomers of dynamin to form via G-domain dimerization, activating the GTPase. CryoEM studies have shown that lipid type, rather than overall lipid charge is important for Drp1 function. When Drp1 was bound to CL, it lay closer to the membrane than when it was bound to PS, which has the same charge (Box 1). It was suggested that the disordered nature of the VD acts to bind and cluster multiple CL molecules, resulting in membrane interactions stabilized by high avidity multivalent interactions. Spatial sorting of CL lipids has been shown to cause local regions of membrane constriction, which are then primed to undergo fission [113–115]. The Drp1–CL interaction is a prime example of a protein–membrane interaction working synergistically to co-ordinate and action signals.

**Box 1.**
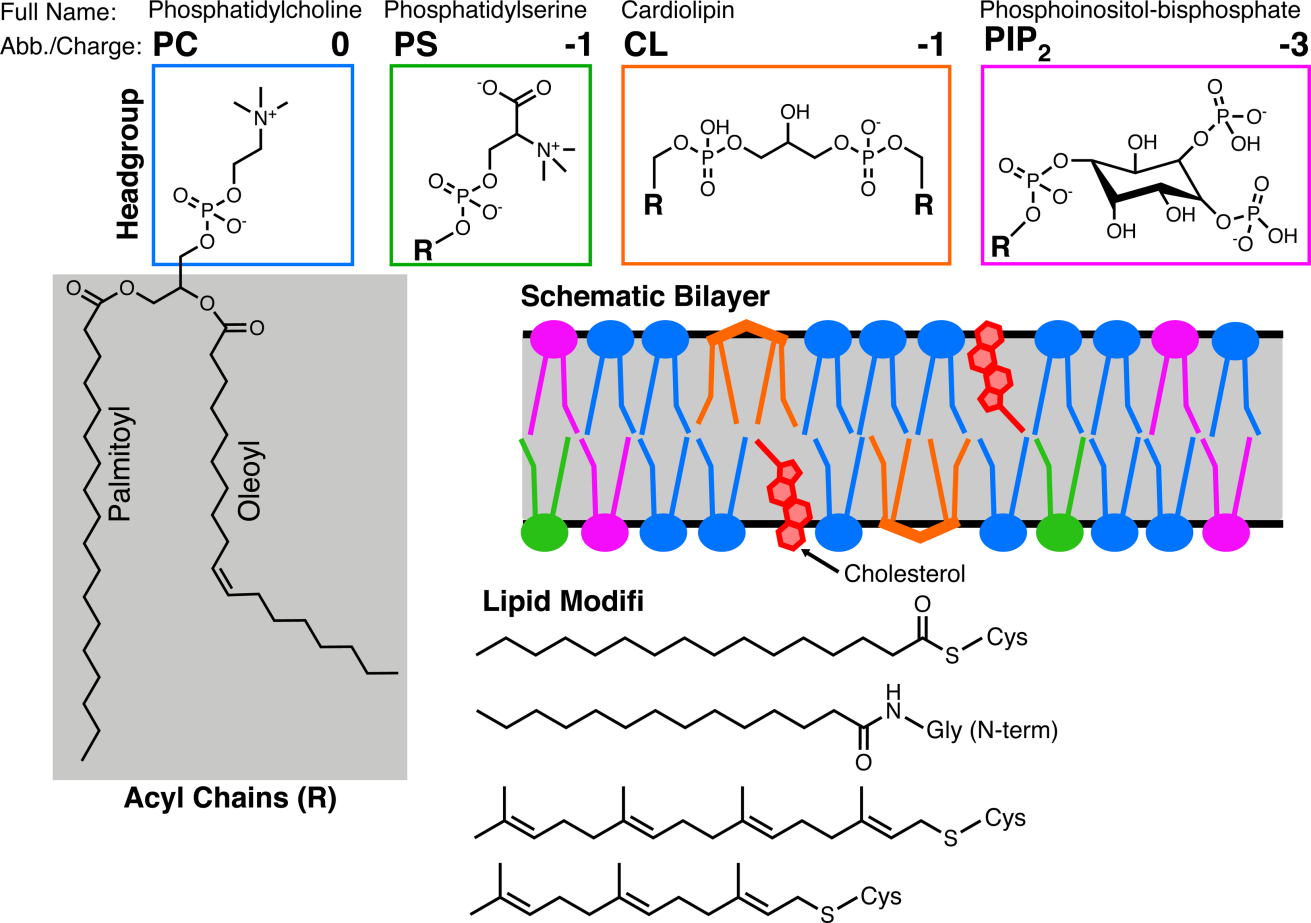
Lipid nomenclature and chemical structures.

## Hypervariable tails in small GTPase signalling

The Ras superfamily of small GTPases are ubiquitous signalling proteins involved in almost all cellular processes (reviewed in [116]). Members of this superfamily contain a highly conserved nucleotide-binding G-domain that adopts a common topological fold with two switch regions that respond to cycling of the bound nucleotide between GDP (inactive) and GTP (active) conformations [117]. The active, GTP-bound conformation can be further subdivided into two conformational states, state 1 (intermediate) and state 2 (active). Many decades of valuable research have shed light on the intricacies of the G-domain. Recently, however, research into the short, intrinsically disordered C-terminal extensions of the Ras, Rho and Rab family proteins has intensified.

**Figure 5.**
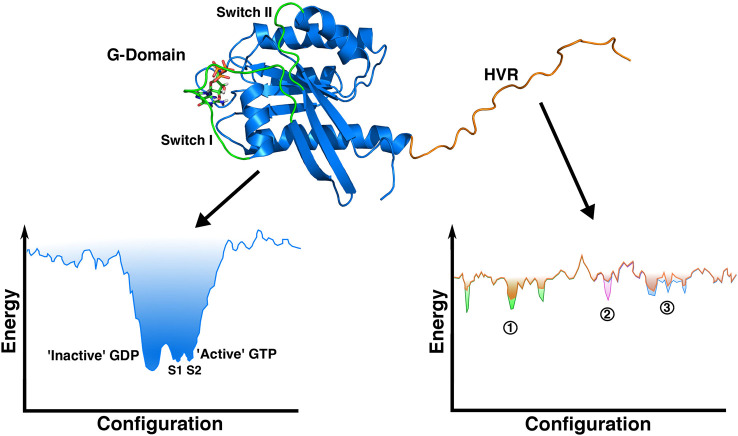
Representation of theoretical energy landscapes of the folded G-domain vs the intrinsically disordered HVR in RalA. The G-domain of RalA is shown in blue (co-ordinates from our unpublished structure), with switch regions shown in green. The HVR of RalA is represented by the ribbon structure in orange. Schematic representations of the energy landscape sampled by each of these regions of the protein are shown below. The G-domain will have a global energy minimum, corresponding to the correctly folded state. Within this global minimum, there will be local minima representing both the GDP and GTP bound forms of the protein corresponding to inactive and active states of the protein respectively. Similar to that of Ras proteins, the active GTP-bound state can be further subdivided into distinct minima for states s1 (intermediate) and s2 (active) conformations. In contrast, the energy landscape sampled by the disordered HVR will be composed of multiple shallow minima separated by small energy barriers. This shallow, rugged energy landscape imbues this region with significant conformational dynamics, interactional promiscuity and sensitivity to environmental changes. Interaction with various regulatory proteins (1, green), post-translational modifications e.g. phosphorylation (2, pink) and interaction with various membrane environments (3, blue) can change the energy landscape by modifying local minima and/or energy barriers between minima, thus changing the structure and dynamics of the HVR.

As with all intrinsically disordered regions, the energy landscape sampled by the hypervariable region is rugged and shallow compared with the deep energy minima experienced by the G-domain (Figure 5). The HVRs of small GTPases are the site of post-translational lipid modification(s), which anchor the protein to the membrane. They are also the site of most sequence variation (hence the name) within small G protein families and potentially hold the key to understanding how even highly related members of subfamilies can display such diverse signalling behaviours, both in normal cellular function and in disease settings.

**Figure 6.**
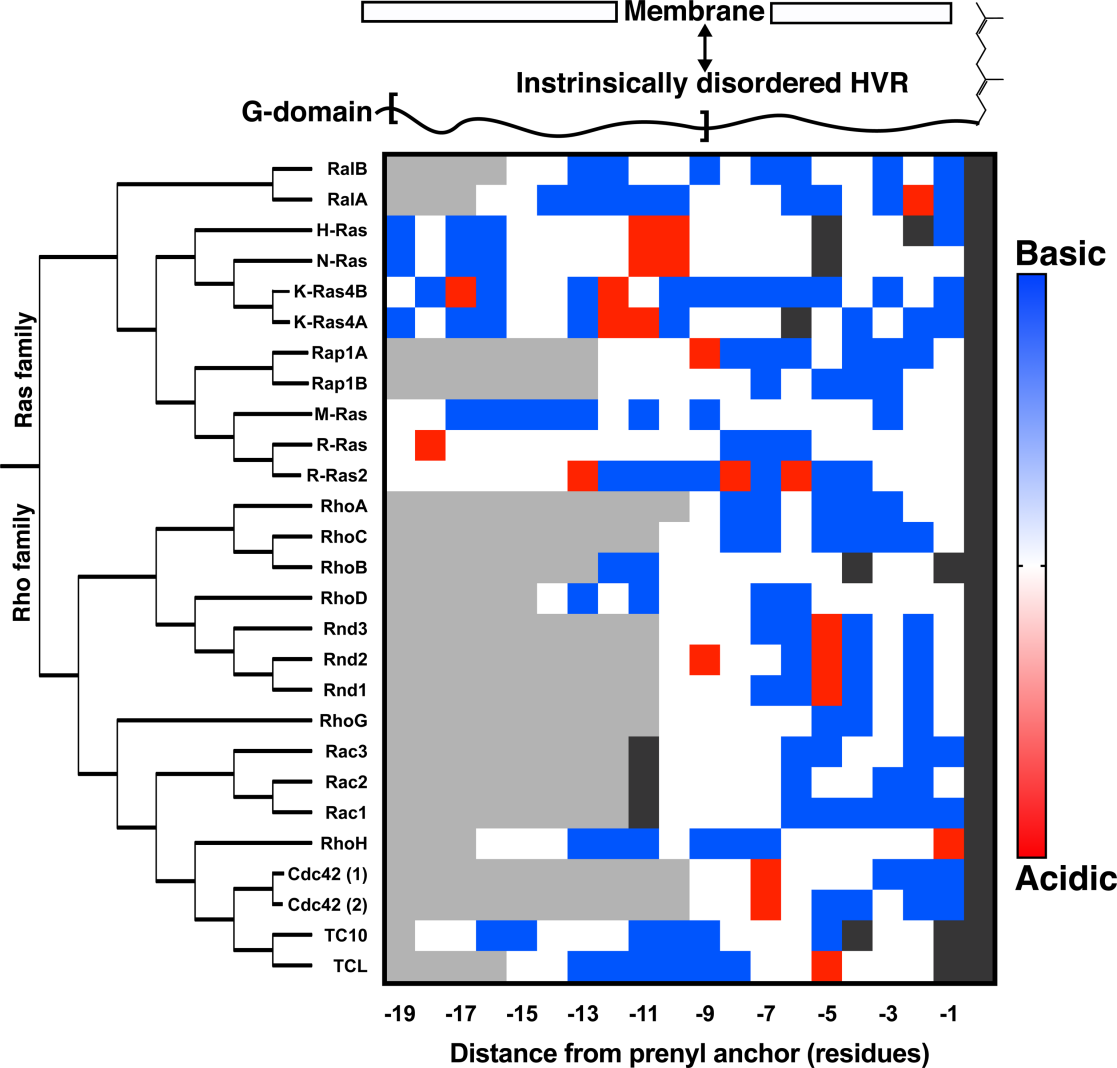
Diversity in hypervariable regions of the Ras and Rho families. Plot showing the distribution of basic and acidic residues in the hypervariable regions of various proteins of the Ras and Rho families. The hypervariable regions of these proteins are characterized by having a high degree of positive charge or further lipid modification sites to aid membrane interaction and association. Sequences are aligned on the terminal isoprenylated cysteine residue (dark grey), residues of the hypervariable regions are coloured by their properties: basic residues are in blue, acidic in red, sites of potential additional lipidation in dark grey and all others are white. Light grey denotes residues of the G-domain. There is a large variation in both sequence and charge distribution within these HVRs, even when comparing highly similar isoforms of a protein e.g. RalA and RalB. This hypervariability led to the conclusion that these disordered regions, once neglected, must be crucial for the complex signalling of these proteins.

The influence of small GTPase HVRs on signalling behaviour and membrane attachment is becoming better understood and many of the principles discussed thus far in this review are demonstrated by these small, intrinsically disordered regions. The HVRs range from 10–20 residues but what they lack in size, they make up for in sequence diversity (Figure 6) and in their ability to bring additional signalling complexity to these simple molecular switches. Small GTPases used to be considered to be ‘balloons on strings’, with their disordered tails merely serving to attach them to biological membranes. Now, however, it is apparent that far from being just a lipidated membrane anchor, these disordered tails are key to proper regulation and function of small GTPases, as well as being an important tool for their localization.

### Localization

Early work with GFP-tagged HVRs suggested that the HVR alone was necessary and sufficient to drive correct localization [118]. Simple electrostatics were thought to be responsible, with mutational analysis of the K-Ras4B HVR suggesting that simply the number of positively charged residues present resulted in correct plasma membrane (PM) localization [42,43]. More recently, it was noted that K-Ras4B maintains its PM localization despite the presence of the vast endomembrane system with a similarly charged lipid composition [119]. An active cyclic process was uncovered, involving the interaction of the HVR and the prenyl anchor with PDE-δ [120] and subsequent release by Arl2 at defined locations, which drives correct K-Ras4B localization [119,121]. Similarly, a motif of acidic residues, conserved between H-Ras, N-Ras and K-Ras4A (Figure 6), is thought to drive specific interactions with acyltransferases, conferring precise localization through palmitoylation [122]. Further regulation is provided through the interaction of depalmitoylated H-Ras with PDE-δ. This interaction increases the efficiency of Golgi trapping and directional secretion back to the PM [123]. Despite the size of the HVR, its intrinsic disorder allows almost a scaffolding effect, integrating membrane interaction motifs with protein–protein interaction motifs to drive microlocalization. For example, Rac1 can co-localize with PACSIN-2 to membrane ruffles, through simultaneous HVR interaction with the PACSIN-2 SH3 domain and with the PM [124,125]. The F-BAR region of PACSIN-2 can then specifically regulate this Rac1 micropool through endosome internalization and subsequent interaction with GAPs (GTPase Activating Proteins), proteins which ‘switch off’ GTPase activity by stimulating their intrinsic hydrolysis of the bound nucleotide [124,125].

### Reorientation of a globular domain at the membrane

There has recently been much focus on how the disordered HVR and G-domain of these proteins co-operate to orientate the G-domain with respect to the membrane and what effect its reorientation might have on signalling. Thanks to ground-breaking work on K-Ras4B and Rheb proteins from the Ikura lab, distinct orientation states were observed with these proteins in complex with membrane-mimicking nanodiscs. Different states were favoured, depending upon nucleotide status or oncogenic mutation, and could be observed by NMR [126,127]. The surprising discovery of a membrane orientation state of K-Ras4B that occluded the binding site for downstream effectors was validated by the development of an inhibitor that stabilized this occluded state [128,129]. More recently this work has been extended to show that homodimer formation of active, GTP-bound K-Ras4B favours a membrane orientated state that promotes effector accessibility [130]. Moreover, it has been suggested that formation of this active signalling dimer facilitates orientation of a ternary complex between the membrane, K-Ras4B and its effector kinase C-Raf, in a manner that enhances successful autophosphorylation of C-Raf [131]. Work on H-Ras corroborates the importance of G-domain membrane orientation. It was found that a region of the G-domain and the HVR co-operate to tune orientation [132–135] and that oncogenic mutations in this element augments nanoclustering and therefore signalling output [133,135]. Extended molecular dynamic (MD) simulations suggest that HVR-membrane interactions impose restraints on the G-domain tilt angle relative to the membrane which results in three orientation states [136]. As different protein–protein interactions are favoured/possible in each orientation, the membrane has the potential to act as an allosteric inhibitor or activator based on only a small change in tilt angle [136]. Dynamics of the HVR may also mediate orientation preference: MD simulations of K-Ras4B showed that the conformational dynamics of the HVR differ in each major orientation state [137]. The energy barriers between membrane orientations are relatively small, so that reorientation can be driven by protein conformational dynamics, followed by stabilizing protein–lipid interactions [138]. A lack of persistent contacts between the HVR and the G-domain of K-Ras4B in MD trajectories [137,139] led to further investigation and the discovery that K-Ras4A, with its highly divergent HVR but similar G-domain, samples similar membrane orientations to that of K-Ras4B. This resulted in the suggestion that it is the topology of the G-domain that drives membrane orientation states rather than the HVR [140]. Indeed, work from the same group showed that even with HVR-truncated K-Ras4B there was some NMR-based evidence for multiple orientations of the G-domain with respect to a membrane mimetic surface [141]. Nevertheless, it seems likely that instead of being solely the HVR or the G-domain that drives membrane orientation, it is the co-operation between these disordered and structured elements at the membrane that drives orientation preference. While the G-domain alone may be capable of supporting each major membrane orientation, the HVR through conformational dynamics [137,138], allosteric [132,135], and direct interactions [142,143], between the HVR and G-domain, and interactions with the membrane that restrict possible tilt angles [136], can tune and modulate the sampling of these membrane orientation states. More recently it has been suggested that these membrane orientated states of K-Ras4B that are proximal to the membrane are only a small subsection of a larger ensemble where most states are membrane distal. Work by Stephen and colleagues has suggested that recruitment of the Ras-effector RAF1 by K-Ras4B could occur in a multi-step fly-casting mechanism. In this proposed mechanism, the membrane co-operates with the HVR and G-domain to orient K-Ras4B in a exposed orientation compatible with effector engagement, the G-domain transiently dissociates from the membrane to engage RAF1 before the HVR facilitates the re-recruitment of the G-domain in complex with RAF1 to the membrane [32].

### Clustering and lipid sorting

The interplay between the disordered tail and membranes does not stop at the regulation of GTPases by the membrane environment and may not be limited to one-way communication. The complex communication between these proteins and their lipid environment was suggested when it was discovered that HVRs can drive the sorting and spatial segregation of lipids [39,144]. This sorting was found to be more complex than simply electrostatic recruitment of lipids, as disordered tails with the same net charge but different sequences resulted in differential lipid sorting profiles [39]. It is suggested that this ‘lipid code’ could be realized, in part, through order to disorder transitions of the HVR. A metadynamic MD simulation of the HVR of K-Ras4B found that it exists in three main populations: disordered, intermediate and ordered conformations, with relatively small energy barriers between these states [39]. The energy landscape of these three states are modified by changes to sequence, type of prenyl moiety and post-translational modifications, such as phosphorylation. The modification of this energy landscape alters lipid preference, probably via the changes in H-bond network that occur upon an order to disorder transition [39,144]. Further work has shown that lateral segregation of PS, which is promoted by the lipid sorting ability of the K-Ras4B HVR, has the effect of nanoclustering K-Ras4B. These higher-order aggregates of the G-protein and specific lipids serve to act as a high fidelity signalling platform, which potentiates signalling while decreasing noise [144,145]. In contrast, H-Ras reduces PS segregation and therefore down-regulates K-Ras4B nanoclustering and signalling. This is mediated solely through the disordered HVR, as the expression of H-Ras HVR alone recapitulates this effect [145]. Furthermore, there is evidence to suggest that the interaction of the disordered tail of K-Ras4B can regulate nanoclustering (and therefore signalling) in response to both membrane potential [146] and membrane curvature [147]. The membrane sensing and modulating ability is driven mainly by the HVR [145,148]. Nanoclustering is likely to be widespread amongst small GTPases and contributes to isoform specific complexity in signalling roles. Nanoclusters of Cdc42 [149] and Rac1 [150,151] have also been demonstrated.

### Post-translational modification

Many small GTPases have phosphorylation sites within their disordered tails. The ability to rapidly modulate the energy landscape of this disordered region allows for additional levels of signalling regulation and complexity to be enacted through the HVR (Figure 5). Phosphorylation is often observed to cause relocation and subsequently change the signalling behaviour of small GTPases, as observed with Rap1A [152], RalA [153] and Wrch1 [154]. This could be a simple electrostatic switch, whereby phosphorylation reduces the charge of the polybasic HVR resulting in destabilization of membrane interactions [152]. Alternatively, phosphorylation could cause a rearrangement of the HVR backbone that either promotes membrane withdrawal by caging of the prenyl anchor [155] or enhances HVR-membrane interactions. However, the effects of phosphorylation are likely to be more complicated and nuanced than these simplistic explanations. It was observed that K-Ras4B undergoes spatial segregation into distinct and non-overlapping membrane clusters depending upon phosphorylation at Ser181 in the HVR and that the phosphorylated K-Ras clusters co-localize with PI3-Kinase [156]. It is possible that phosphorylation changes the dynamics of the HVR, modulating the lipid sorting preference for PIP_2_ and PIP_3_ lipids [39], which then mediates the formation of K-Ras4B/PI3-Kinase signalling platforms.

**Figure 7.**
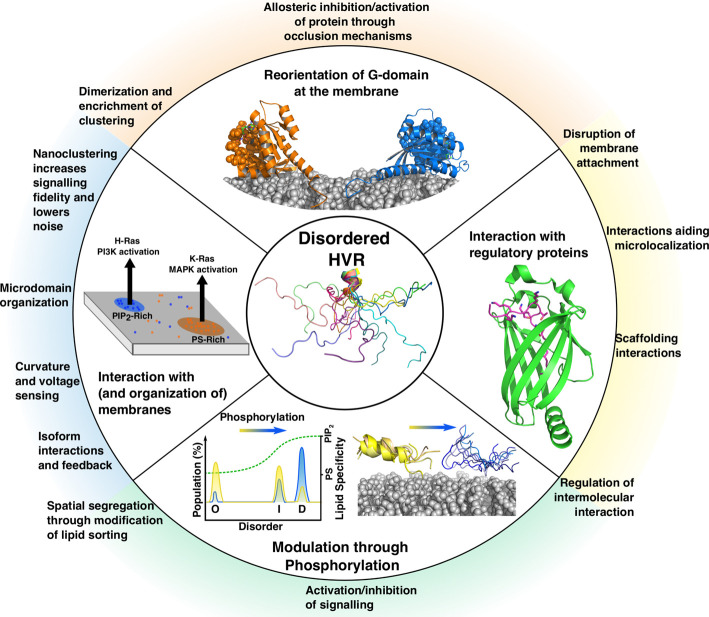
Summary of how the HVR modulates the signalling of small GTPases. The inner circle shows an HVR adopting a number of conformations, the middle circle represents mechanisms whereby the HVR can modulate signalling and the outer circle describes how this modulation can affect signalling. Middle circle: (Top) Models of K-Ras-4B·GDP (in orange) K-Ras-4B·GTP (in blue) interacting with the surface of a nanodisc, PDB codes 2MSC, 2MSD, respectively [127]. The effector binding region is shown as spheres: with the change in nucleotide there is a change in orientation with respect to the membrane. (Right) Interaction of the HVR of K-Ras-4B (magenta) with PDE-δ (green), PDB code 5TB5 [120]. (Bottom) Schematic representing the change in dynamics of the HVR of K-Ras-4B in response to phosphorylation at Ser181 as proposed by Zhou and colleagues [39].The unmodified HVR exists as a population with varying degrees of disorder (O = ordered, I = intermediate, D = disordered), phosphorylation causes a shift in population to the disordered state which favours interaction with PIP_2_ lipids instead of phosphatidylserine (PS). (Left) Schematic showing the macroorganization of Ras proteins into discrete membrane domains, which results from the differential sorting abilities of the HVR. K-Ras-4B (orange dots) cluster in domains enriched with PS lipids to potentiate MAPK signalling through C-Raf activation, while H-Ras (blue dots) clusters into separate domains enriched in PIP_2_ and increases signalling through PI3K.

It is generally accepted that disorder in proteins aids promiscuity of binding [157]. This promiscuity allows the interaction of the HVR of GTPases with an array of proteins that regulate localization and function such as Calmodulin [158,159], PDE-δ [120], Galectin [160,161] and PACSIN-2 [124] but also the varied and transforming landscape of distinct membranes. Rather than being simple electrostatic anchors, the sequence of each HVR dictates the conformational dynamics that these regions explore and their energy landscapes (Figure 5) make the HVR very sensitive to environmental change [157]. This is key for both the two-way cross-talk between the membrane and the HVR, as well as the ability of post-translational modifications, such as phosphorylation, to tune and regulate this crosstalk [162]. HVRs generally remain disordered while contributing to protein localization, G-domain reorientation, membrane sensing, modulation and nanoclustering as well as interacting with regulatory proteins [124,158–160] (Figure 7). The lack of global fold has allowed greater sequence space to be explored during evolution [157], contributing to the functional diversity displayed by the abundant and closely related members of the Ras superfamily.

## Conclusion

This review has focussed on the interplay between disordered regions and the membrane and how this dynamic cross-talk is integral to cell signalling. Disorder plays a role in membrane modulation, lipid and curvature sensing, clustering and phase separation events, transduction of signal across membranes, protein scaffolding and in the coupling of enzymatic activity to these processes. Through conformational plasticity, a single disordered region or multiple disordered modules can co-operate with structured domains to provide layers of spatiotemporal regulation within cell signalling. Our discussion of the HVR of small GTPases highlights how intensive study of these disordered regions can enrich understanding of cell signalling as a whole. Far from being a marriage of convenience, intrinsically disordered regions and membranes are a match made in heaven for cell signalling.

## Perspectives

- Intrinsically disordered proteins and regions, which are prevalent across biology, act as crucial mediators of cell signalling. Biological membranes, which act to compartmentalize biological processes, often act in harmony with disordered regions to expand and modify the function of folded domains involved in cell signalling.
- Co-operation between disordered proteins and membranes can provide nuanced modulation to cellular signalling events, often through spatiotemporal regulation of signalling complexes.
- An understanding of the complexity and diversity within cell signalling will require greater attention to be focused not just on folded domains but the disordered regions which often flank them. Integrated approaches, which combine classical structural, biophysical and cell biology experiments with techniques able to assess the contribution of disordered regions in the context of a membrane environment, such as NMR and MD simulations, will promote a more holistic understanding of the proteins involved in cell signalling and the pathways in which they sit.

## Open Access

Open access for this article was enabled by the participation of University of Cambridge in an all-inclusive *Read & Publish* pilot with Portland Press and the Biochemical Society under a transformative agreement with JISC.

## Abbreviations

ALPS: amphipathic lipid packing sensor
CL: cardiolipin
CryoEM: cryogenic electron microscopy
EGF: epidermal growth factor
EGFR: epidermal growth factor receptor
GAP: GTPase activating protein
GDP: guanosine diphosphate
GFP: green fluorescent protein
GTP: guanosine triphosphate
HVR: hypervariable region
IDP: intrinsically disordered protein
IDR: intrinsically disordered region
ITAM: immunoreceptor tyrosine-based activation motif
JM: juxtamembrane
LAT: linker for activation of T-cells
MD: molecular dynamics
NMR: nuclear magnetic resonance
PIP_2_: phosphatidylinositol (4,5) bisphosphate
PIP_3_: phosphatidylinositol (3,4,5) triphosphate
PS: phosphatidylserine
PTM: post-translational modification
VD: variable domain
